# Identification and functional characterization of the first deep intronic *GLA* mutation (IVS4+1326C>T) causing renal variant of Fabry disease

**DOI:** 10.1186/s13023-022-02377-8

**Published:** 2022-06-20

**Authors:** Xuantong Dai, Xue Zong, Xiaoxia Pan, Wei Lu, Geng-Ru Jiang, Fujun Lin

**Affiliations:** 1 Renal Division, Department of Internal Medicine, Xin Hua Hospital, Shanghai Jiao Tong University School of Medicine, Shanghai, 200092 China; 2 Department of Nephrology, Ruijin Hospital, Shanghai Jiao Tong University School of Medicine, Shanghai, 200020 China; 3 Centre for Rare Disease, Shanghai, 200092 China

**Keywords:** Fabry disease, Renal variant, Podocyte, *GLA* gene, Deep intronic mutation, α-Galactosidase A

## Abstract

**Background:**

Fabry disease (FD, OMIM #301500) is an X-linked lysosomal disorder caused by the deficiency of α-galactosidase A (α-GalA), encoded by the *GLA* gene. Among more than 1100 reported *GLA* mutations, few were deep intronic mutations which have been linked to classic and cardiac variants of FD.

**Methods and results:**

We report a novel hemizygous deep intronic *GLA* mutation (IVS4+1326C>T) in a 33-year-old Chinese man with a mild α-GalA deficiency phenotype involving isolated proteinuria and predominant globotriaosylceramide deposits in podocytes. IVS4+1326C>T, which appears to be the first deep intronic *GLA* mutation associated with renal variant of FD, was identified by Sanger sequencing the entire *GLA* genomic DNA sequence of the patient’s peripheral mononuclear blood lymphocytes (PBMCs). Further sequencing of cDNA from PBMCs of the patient revealed a minor full-length *GLA* transcript accounting for about 25% of total *GLA* transcript, along with two major aberrantly spliced *GLA* transcripts encoding mutant forms of α-GalA with little enzyme activity characterized by in vitro α-GalA overexpression system in the HEK293T cells. Thus, the combined clinical phenotype, genetic analysis and functional studies verified the pathogenicity of IVS4+1326C>T.

**Conclusions:**

The identification of IVS4+1326C>T establishes a link between deep intronic *GLA* mutation and the renal variant of FD, which extends the mutation spectrum in *GLA* gene and justifies further study of how IVS4+1326C>T and potentially other deep intronic *GLA* mutations contribute to Fabry podocytopathy through aberrant splicing. Future studies should also assess the true incidence of IVS4+1326C>T in patients with different variants of FD, which may improve early genetic diagnosis to allow timely treatment that can prevent disease progression and improve survival.

**Supplementary Information:**

The online version contains supplementary material available at 10.1186/s13023-022-02377-8.

## Introduction

Fabry disease (FD) is a rare X-linked lysosomal disorder characterized by deficiency of α-galactosidase A (α-GalA), encoded by the *GLA* gene. α-GalA deficiency results in intracellular accumulation of neutral glycosphingolipids, primarily globotriaosylceramide (GL-3) and its deacylated derivative globotriaosylsphingosine (Lyso-GL-3) inside of lysosomes, leading to multi-organ involvement. Fabry nephropathy is a hallmark of FD and in untreated FD patients of classic phenotype with absent or negligible α-GalA activity (< 1% residual enzyme activity, REA), end stage renal disease (ESRD) usually occurs by the time they reach their 40 s [1] if the disease goes untreated with enzyme replacement therapy (ERT) with α-GalA or chaperone therapy, the early initiation of which can slow or stop renal deterioration.

Screenings for FD have shown that FD prevalence in male patients with chronic kidney disease (CKD) or ESRD is 0.48–1.69% [2]. Thus, FD needs to be considered in the differential diagnosis of any patient with unexplained CKD, as FD can present as ‘renal variant’, with renal disease as the only or prominent manifestation [3]. Having varying levels of residual α-GalA activity, FD patients with renal variants usually lack glycosphingolipid accumulation in microvascular endothelium and thus the classic manifestations such as angiokeratoma, acroparesthesia and hypohydrosis are absent, which often leads to a delay in diagnosis.

Mutation analysis of the *GLA* gene is a valuable tool for screening and diagnosis of FD. Renal variant of FD reported so far has been linked to numerous *GLA* missense mutations (p.Ala37Thr [4]; p.Met42Leu [5]; p.Glu66Lys [6]; p.Glu66Gln [3]; p.Ile91Thr [4]; p.Arg112His [4, 7]; p.Phe113Leu [8]; p.Ala143Thr [9]; p.Arg196Thr [4]; p.Pro205Ser [4]; p.Pro210Ser [10]; p.Phe229Val [11]; p.Met290Val [4]; p.Arg356Gly [4]; p.Gly360Ser [12]) and two frameshift mutations (p.Leu344fs*31 [13]; p.Lys426Argfs*24 [14]). In this study, we reported the identification of a novel deep intronic *GLA* mutation (IVS4+1326C>T) in a 33-year-old Chinese man with a mild α-GalA deficiency phenotype involving isolated proteinuria and predominant GL-3 accumulation in podocytes. IVS4+1326C>T, which appears to be the first deep intronic *GLA* mutation associated with renal variant of FD, was identified by analyzing the full length *GLA* genomic DNA sequence from the peripheral mononuclear blood lymphocytes (PBMCs) of the patient. Molecular studies were further performed to investigate how IVS4+1326C>T may contribute to renal variant of FD.

## Methods

### Participants and ethical statement

The proband patient was admitted to Xin Hua Hospital in 2018 with proteinuria two years. Written informed consent was obtained from the involved subjects and all procedures were performed in accordance with the Declaration of Helsinki. The study was performed under the approval of the Ethics Committee from Xin Hua Hospital, Shanghai Jiao Tong University School of Medicine (approval No.XHEC-D-2020-002).

### DNA and RNA isolation

Blood samples were collected from the patient, his family members and normal controls using test tubes containing EDTA anticoagulant. Genomic DNA was extracted from PBMCs using DNA Midi Kit (Qiagen, Milano, Italy) according to the manufacturer’s protocol. Total RNA in PBMCs was collected in Tempus™ blood RNA tubes (Biotek, Canada) and isolated with Tempus™ Spin RNA Isolation kit (Biotek, Canada) according to the manufacturer’s protocol.

### Genomic DNA sequencing analysis

The full length genomic DNA of *GLA* were amplified by nested polymerase chain reaction (PCR). Primers are designed according to the genomic *GLA* reference sequence (NCBI reference sequence NM_000169) listed in Additional file 1: Table 1. Sanger sequencing was performed to verify the sequences.

### Whole exome sequencing (WES)

Whole-exome sequencing was performed on the patient as described [15], and the WES data were evaluated for causative mutations in *GLA* and other genes associated with inherited podocytopathies [16]. The non-neutral variant p.Arg229Gln in the *NPHS2* gene and the high-risk alleles G1 and G2 in the *APOL1* gene were manually inspected.

### Reverse transcription polymerase chain reaction (RT-PCR) and quantitative RT-PCR (qRT-PCR)

To evaluate the range of transcripts produced from the *GLA* gene, total RNA was extracted from PBMCs from the patient, his mother, and healthy controls as described above. First-strand cDNA synthesis was performed using the Hifair™ 1st Strand cDNA Synthesis SuperMix Kit for qPCR (Yeasen, Shanghai, China). Then PCR was performed using the PrimerSTAR MAX DNA Polymerase (TaKaRa, Beijing, China), and amplicons were visualized on 1.5% agarose gels and sequenced on an ABI PRISM 3100 Genetic Analyzer. Primer sequences were listed in Additional file 1: Table 1.

To quantify the effect of IVS4+1326C>T on the expression of *GLA* mRNA level in PBMCs, qRT-PCR were performed using 2 pairs of cross-exon primers (listed in Additional file 1: Table 1) and Green Master Mix (Yeasen, Shanghai, China) to specifically amplify the full-length transcript of *GLA*, and qRT-PCR was normalized to that of GAPDH. Data was analyzed using software Geneious version 10.2.3 (Biomatters, Auckland, New Zealand).

### Plasmid construction

Full-length human *GLA* cDNA was subcloned into the PHAGE vector at the *Bam*HI and *Sal*I sites, upstream from a hemagglutinin (HA) tag. The resulting PHAGE-*GLA* plasmid was then subjected to multiple PCR using mutated primers (listed in Additional file 1: Table 1) and PrimerSTAR MAX DNA Polymerase (TaKaRa Bio, Beijing, China) to give rise to mutant PHAGE-*GLA* plasmids encoding aberrantly spliced transcripts of *GLA*. All constructs were verified by sequencing.

### Cell culture and transfection

HEK293T cells were maintained in DMEM supplemented with 10% FBS, 100 U/ml penicillin and 100 mg/ml streptomycin at 37 °C with 5% CO_2_. HEK293T cells were transfected with the wild-type PHAGE-*GLA* plasmid or mutant PHAGE-*GLA* plasmids using Lipofectamine 2000 (Invitrogen, California, USA) according to the manufacturer’s instructions. Transfected cells were incubated for 24–48 h post transfection and proteins were analyzed by Western blot analysis.

### Western blot analysis

Cell lysates were prepared using Cell Lysis Buffer (Beyotime, Shanghai, China). Protein (10–40 µg) was fractionated on 8–12.5% SDS–polyacrylamide gels and transferred to 0.22-µm polyvinylidene fluoride membranes. After 1 h of incubation at room temperature in 5% milk, the membrane was incubated with the primary antibody for 2 h followed by incubation with the appropriate secondary antibodies for 2 h. Antibody binding was detected using the ECL Plus Western Blotting System (GE, Buckinghamshire, UK). GAPDH was detected as an internal control. The primary antibodies were mouse monoclonal anti-HA antibody (1:2000, Cell Signal Technology, Massachusetts, USA) and rabbit polyclonal anti-GAPDH antibody (1:1000, Cell Signal Technology, Massachusetts, USA).

### α-Galactosidase (α-GalA) enzyme assay

Activities of α-GalA in cell lysates of HEK293T cells transfected with the wild-type or mutant PHAGE-*GLA* plasmids were measured using the Micro α-Galactosidase Assay Kit (Solarbio, Beijing, China) according to the manufacturer’s instructions. To correct for endogenous α-GalA activity in HEK293T cells, a mock control plasmid was also transfected and the level of α-GalA activity in lysates from these cells were subtracted from those in mutant- or wild-type-transfected cells.

### Statistical analysis

Continuous data that were normally distributed were expressed as mean ± standard deviation. Intergroup differences were assessed for significance using ANOVA with Bonferroni-Holm post-test correction or Student’s *t* test, as appropriate. Differences associated with *P* < 0.05 were considered statistically significant.

## Results

### Clinical description

A 33-year-old Chinese man was referred to Xin Hua Hospital in 2018 with proteinuria two years prior to renal biopsy. On admission, his blood pressure and pulse rate were in the normal range. Laboratory data on admission showed that the serum creatinine level was 0.85 mg/dL, blood urea nitrogen level was 15 mg/dL and 24-h proteinuria was 1100 mg. The following serum test results were normal or negative: complement testing, assay for anti-neutrophil cytoplasmic antibody, assay for anti-DNA antibody, hepatitis serology, and protein electrophoresis.

**Fig. 1:**
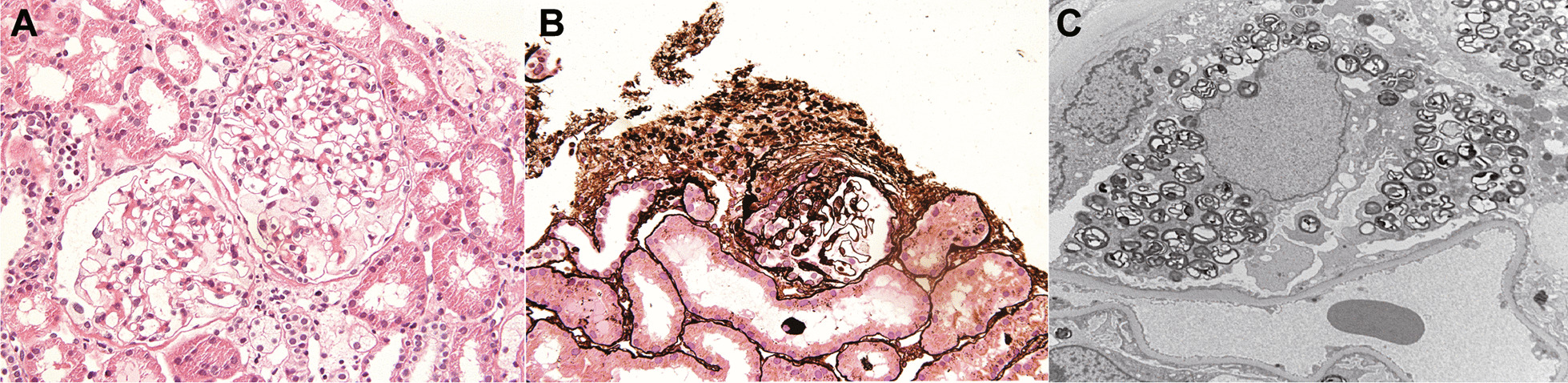
**A–C** Kidney biopsies of the patient. **A** Tissue was stained with periodic acid-Schiff reagent, revealing two glomeruli with enlarged and vacuolated podocytes. Magnification, 400×. **B** Tissue was stained with periodic acid silver methenamine, showing one glomerulus with segmental sclerosis. Magnification, 400×. **C** Electron micrograph showing abundant, electron-dense myelin structures within the cytoplasm of podocytes. Magnification, 5000×

A renal biopsy was performed and eight glomeruli were observed by light microscopy. Global sclerosis was identified in two glomeruli, while the other six showed foamy changes in the podocytes (Fig. 1A), and one glomerulus had segmental sclerosis (Fig. 1B). Vacuolation was present in some tubular epithelial cells, 10% of which showed tubulointerstitial atrophy. No significant alterations in blood vessels were observed. Immunofluorescence findings were unremarkable. Electron microscopy revealed myelin-like inclusions predominantly in the cytoplasm of podocytes (Fig. 1C) with 40% foot process fusion rate. The patient denied ever taking any medicine affecting the activity of lysosomal enzymes, such as amiodarone, chloroquine or tamoxifen and he also denied pain in the extremities, acroparesthesia, hypohydrosis, angiokeratomas, or gastrointestinal disturbance. Measurement of α-GalA activity in patient peripheral blood leukocytes revealed reduced α-GalA activity (27.9 nmol/mL/h/mg; normal, > 37.0 nmol/mL/h/mg). Plasma Lyso-GL-3 was elevated (2.49 ng/mL; normal, 0.24–0.86 ng/mL).

### Genetic analysis

**Fig. 2:**
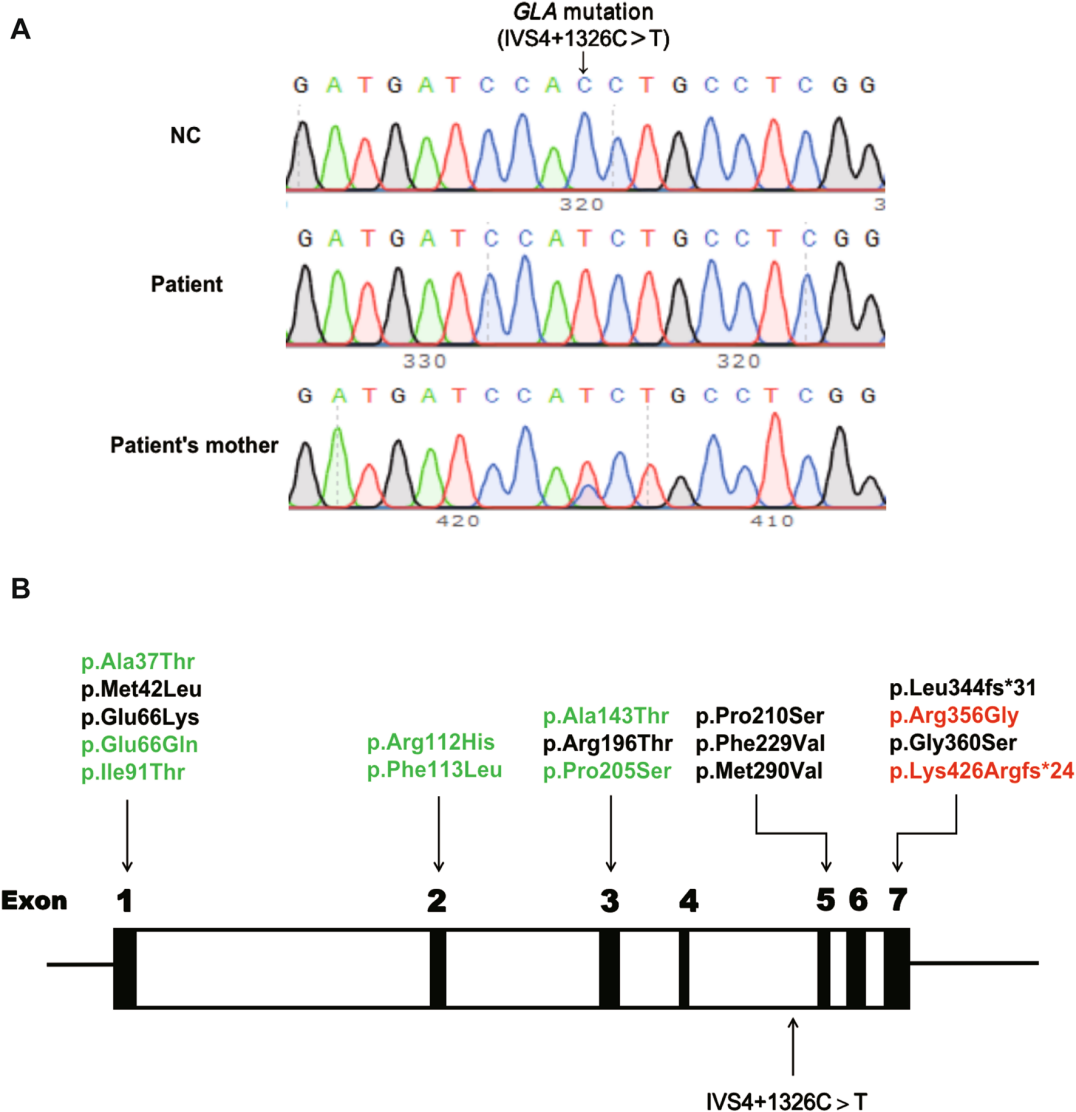
**A** Genomic sequencing of the patient, his mother and a normal control (NC). The patient was hemizygous and the mother heterozygous for the *GLA* mutation IVS4+1326C>T. **B** Schematic of the *GLA* gene indicating the relative position of the seven exons and showing the position of IVS4+1326C>T and the other 19 renal variant associated hemizygous *GLA* mutations. We only include renal variant associated *GLA* mutations reported in the database of PubMed with thorough investigation of all patient organs. Mutations identified only in renal variant of FD are shown in black letters. Mutations also identified in cardiac variant of FD are shown in green letters (p.Ala37Thr [17]; p.Glu66Gln [18]; p.Ile91Thr [19]; p.Arg112His [20]; p.Phe113Leu [21]; p.Ala143Thr [20]; p.Pro205Ser [22]). Mutations also identified in FD patients with classical phenotype are shown in red letters (p.Arg356Gly [23]; p.Lys426Argfs*24 [24])

As the clinical and pathological findings of the patient highly suspicious for Fabry podocytopathy, we performed WES using DNA from the patient's PBMCs to look for mutation in the exonic and flanking intronic regions of *GLA* as well as other genes associated with genetic podocytopathy without identifying any candidate variant. Next, the full length *GLA* genomic DNA from the patient's PBMCs were amplified by nested PCR followed by Sanger sequencing, which revealed a rare hemizygous mutation at nucleotide 9738 (IVS4+1326C>T) (Fig. 2A). IVS4+1326C>T has not been registered in the established FD-specific database such as International Fabry Disease Genotype–Phenotype Database (dbFGP) or broader genetic variants databases such as Human Gene Mutation Database (HGMD), ClinVar Database or Leiden Open Variation Database (LOVD). Thus, IVS4+1326C>T appeared to be the first deep intronic *GLA* mutation associated with renal variant of FD as all other renal variant associated hemizygous *GLA* mutations identified in PubMed were located in the exonic regions of *GLA* gene (Fig. 2B). Family cascade genotyping revealed a heterozygous IVS4+1326C>T mutation in the patient’s mother (Fig. 2A), who did not present any FD-related symptoms or positive laboratory findings at the age of 55. The patient’s younger brother and sister didn’t have the IVS4+1326C>T mutation and we didn’t manage to find other male or female patient relatives from the patient’s maternal side.

**Fig. 3:**
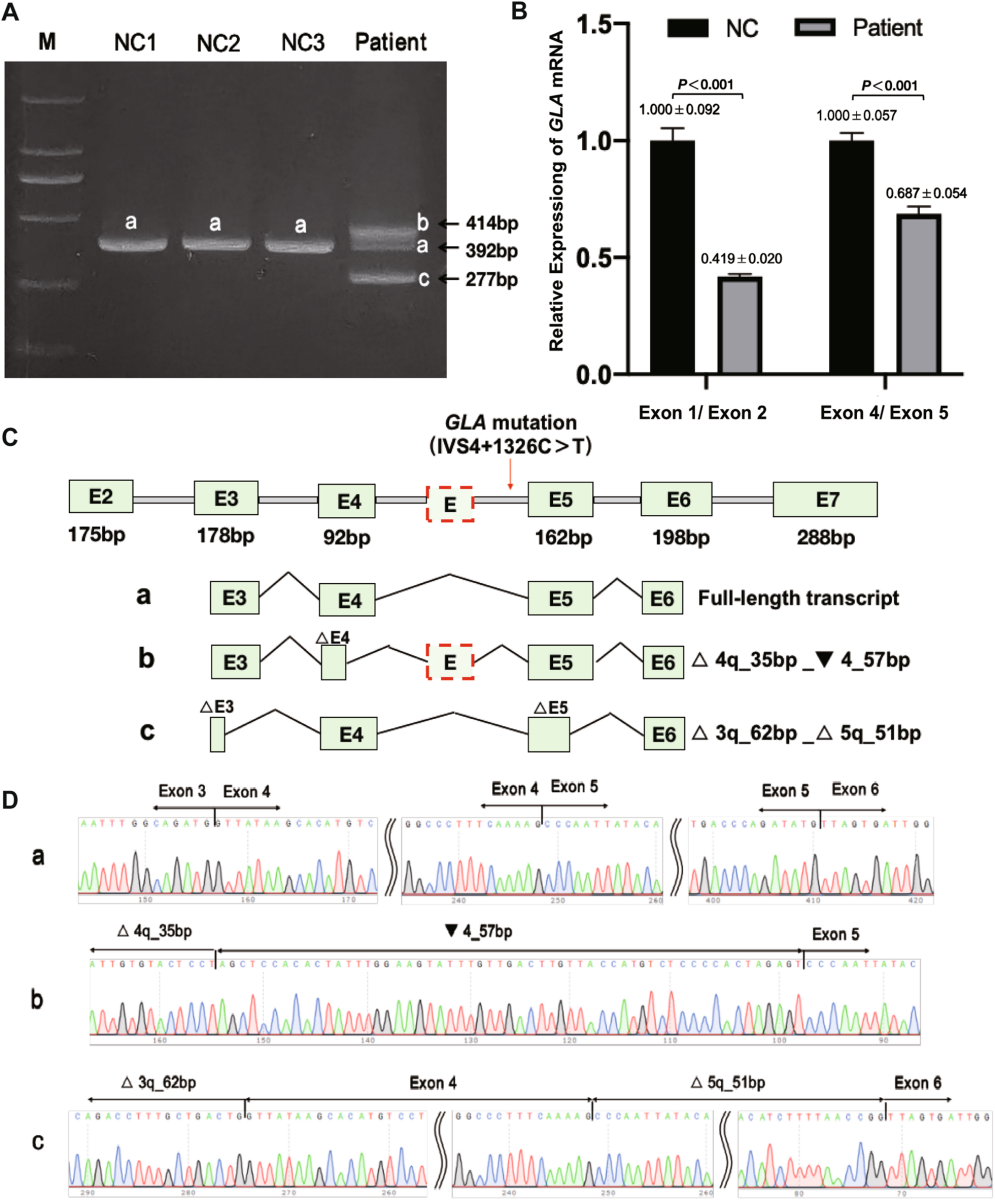
**A** Reverse transcription-PCR analysis of mRNAs from peripheral blood leukocytes of three normal controls (NC1-3) and the patient. In addition to a minor expression of the full length *GLA* transcript (a: 392 bp), the patient showed two major aberrant *GLA* transcripts (b: 414 bp and c: 277 bp). The relative ratio of the three transcripts was quantified by means of examing their grey scale using ImageJ (http://imagej.nih.gov/ij/). **B** qRT-PCR analysis using 2 pairs of cross-exon primers were performed on total RNA from peripheral blood leukocyte of three normal controls and the patient for the quantification of *GLA* full-length mRNA transcript. Levels were normalized to the amount of GAPDH. Data represented the mean ± SD of triplicate experiments. **C** Schematic of normal and aberrant splicing patterns. **D** Sanger sequencing of the full-length 392-bp transcript (a), the 414-bp aberrant transcript (b) and the 277-bp aberrant transcript (c). E: 57-bp pseudoexon; M: molecular weight markers; q: donor-site shift followed by the number of bp that are skipped or included; wt: wild type; △x: skipping of all or part of exon x; ▼: inclusion of intronic sequence in transcript

RT-PCR of *GLA* exons 3 and 6 in mRNAs from the patient’s PBMCs revealed a minor full-length *GLA* transcript (392 bp) as well as two major aberrant splicing transcripts of 414 bp and 277 bp (Fig. 3A). The relative ratio of the three transcripts was 1:1.6:1.4. qRT-PCR showed that the level of full-length *GLA* mRNA from PBMCs of the patient was significantly reduced compared to that of normal controls (Fig. 3B). Further cDNA sequencing showed that the longer aberrant transcript lost a 35-bp sequence at the 5’ end of exon 4 as well as incorporated the 57-bp pseudoexon from intron 4 [25] (△4q_35bp_▼4_57bp), resulting in a premature stop codon (PTC) at residue 202 (p.Cys202*). The shorter aberrant transcript lost a 62-bp sequence at the 5’ end of exon 3 and a 51-bp sequence at the 5’ end of exon 5 (△3q_62bp_△5q_51bp), resulting in a frameshift that generated a PTC at residue 164 (p.Gly163Leufs*2) (Fig. 3C, D).

### Functional experiments

**Fig. 4:**
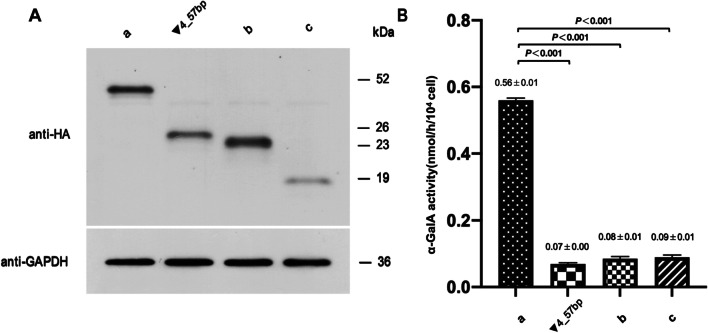
**A** Western blot analysis of cell lysates of HEK293T cells transfected with mutant PHAGE-*GLA* constructs containing △4q_35bp_▼4_57bp (b) or △3q_62bp_△5q_51bp (c). Expression of wild-type PHAGE-*GLA* construct (a) or the mutant PHAGE-*GLA* construct containing ▼4_57bp in HEK293T cells was performed as controls. A hemagglutinin (HA) monoclonal antibody was used for the detection of α-GalA. GAPDH was used as the loading control. The wild-type and mutant protein of 52, 26, 23 and 19 kDa were detected. **B** α-GalA activity (nmol/h/10^4^ cells) in HEK293T cells transfected with wild-type or mutant PHAGE-*GLA* constructs was expressed as the mean ± SD of triplicate experiments. A mock control plasmid was also transfected and the level of α-GalA activity in lysates from these cells (0.09 ± 0.01 nmol/h/10^4^ cell) were subtracted from those in mutant- or wild-type-transfected cells

To further assess the activity of the proteins encoded by the two aberrantly spliced *GLA* transcripts produced by IVS4+1326C>T, we transfected the PHAGE-*GLA* mutant constructs containing △4q_35bp_▼4_57bp or △3q_62bp_△5q_51bp into HEK293T cells in culture. Wild-type PHAGE-*GLA* construct and the mutant PHAGE-*GLA* construct only containing ▼4_57bp were transfected as controls. The full length and mutant protein bands of 52, 26, 23 and 19 kD were detected by western blot analysis (Fig. 4A). The α-GalA activities of HEK293T cells transfected with mutant PHAGE-*GLA* constructs containing the aberrantly spliced transcripts produced by IVS4+1326C>T were less than 20% of that of wild-type PHAGE-*GLA* construct transfected cells (Fig. 4B).

### Treatment and follow-up

Based on the combined kidney biopsy findings, biochemical and molecular analysis, as well as the lack of other FD-related organ involvement as ophthalmological examination, echocardiography, and brain magnetic resonance imaging showed no evidence of FD, the patient was diagnosed with a renal variant of FD. Thus, the patient began taking 75 mg irbesartan per day. Enzyme replacement therapy was not covered by the patient’s medical insurance and so was not provided. At the most recent follow-up, nearly three years after diagnosis, the patient showed normal renal function and 24-h proteinuria of 0.8–1.0 g.

## Discussion

Among more than 1100 *GLA* mutations described in the HGMD, few were intronic mutations that are ‘deep’, i.e. located more than 20 bp away from the exon–intron junction [25–28]. It is presumed that ‘missing mutations’ might reside within the regions that are not covered by routine exonic DNA sequencing whereas sequencing the entire *GLA* gene sequence using traditional Sanger sequencing or the high-throughput sequencing technology such as long-read sequencing nanopore sequencing [27] may help their detection. Besides, deep intronic *GLA* mutations sometimes can be difficult to detect from RNA samples if insufficient RNA is available or if aberrant RNAs are unstable and have been degraded. IVS4+1326C>T identified in our study, to the best of our knowledge, is the first association between the deep intronic *GLA* mutation and renal variant of FD.

Recent studies have supported the pivotal role of podocyte in the development and progression of Fabry nephropathy. Among all renal cell types, GL-3 mainly accumulated in podocytes, which have the slowest turnover rate of the renal cell populations and GL-3 deposits already exist in the majority of normoalbuminuric young classic Fabry patients and was shown to increase with age and directly correlated with proteinuria [29]. Besides, podocytes are relatively resistant to ERT as GL-3 deposits can persist post ERT [30]. In vitro studies generating transient and stable *GLA* knockout FD podocyte cell lines [31, 32] have shown dysregulated cellular signaling pathways in human podocytes. IVS4+1326C>T identified in our case was associated with mild α-GalA enzyme deficiency that manifested predominantly as Fabry podocytopathy. One recent study also found that slightly decreased α-GalA activity may be associated with patients with focal segmental glomerulosclerosis [33]. However, residual α-GalA activity analyzed in peripheral leukocytes of our patient of 75% of normal activity was much higher than the cut-off value for defining a ‘mild’ *GLA* mutation (≥ 20% of mean normal level of α-GalA activity) [34]. Although such high REAs were also observed in other renal variant patients associated with *GLA* missense mutations (Met42Leu [5]; Pro210Ser [10]) and the renal involvement was limited to podocyte pathology, the true threshold level of REA below which could induce podocyte injury remains to be elucidated and the identification of other patients carrying IVS4+1326C>T could help further verify the mild α-GalA deficiency phenotype of Fabry podocytopathy caused by IVS4+1326C>T.

The high residual α-GalA activity associated with IVS4+1326C>T may attribute to the expression of a minor full-length *GLA* transcript as well as the two aberrantly spliced *GLA* transcripts encoding deficient α-GalA as our in vitro study found that each had about 25% of expressed wile-type α-GalA activity. More severe enzymatic phenotypes were observed for two previously reported deep intronic *GLA* mutations in intron 4. IVS4 + 919G > A was first described in a Japanese man with the late-onset cardiac variant of FD [25], and it was later found to be prevalent among Taiwanese patients with hypertrophic cardiomyopathy [35]. IVS4 + 919G > A lies close to the 5’ splice site of the *GLA* pseudoexon, so it disrupts the binding of the nuclear ribonucleoproteins A1 and A2/B1 to the exon splicing silencer that overlaps with the 5’ splice site and that normally prevents the 57-bp pseudoexon inclusion [36]. Consequently, IVS4 + 919G > A induces the overexpression of an aberrantly spliced *GLA* mRNA (less than 5% of the total *GLA* mRNA in normal controls whereas up to 90% of the total *GLA* mRNA in patient carrying IVS4 + 919G > A) that retains the 57-bp pseudoexon, which introduced a PTC predicted to code for a shorter protein devoid of enzyme activity. The remainder of the normal *GLA* mRNA was transcribed to result in about 10% of normal α-GalA activity. Later on, IVS4 + 861C > T was identified in an Italian family presenting with the classic phenotype of FD and nearly no REA [26]. IVS4 + 861C > T lies 5 bp upstream of the alternative 3’ splice site of the 57-bp pseudoexon, and it is predicted to create a new exon splicing enhancer that overexpresses the aberrant transcript containing the 57-bp pseudoexon predominantly and produces negligible full-length *GLA* transcript. The three deep intronic *GLA* mutations with prominent clinical heterogeneity highlight the contribution of aberrant splicing of *GLA* transcripts to FD and also implies that approaches to restore normal splicing of *GLA* transcripts may benefit patients carrying deep intronic *GLA* mutations.

Patients diagnosed with renal variant of FD have variable speed of renal progression. Some arrive at ESRD already in their 40 s, such as those with FD involving the mutations p.Phe113Leu [8] or p.Ala143Thr [9] while others may presented as isolated proteinuria detected by accident at an advanced age, such as patients with FD involving mutations p.Met42Leu [5] or p.Phe229Val [11]. Genotype and phenotype variations also manifest in that *GLA* mutations associated with renal variant of FD have been identified in FD patients with the cardiac variant or the classic phenotype (Fig. 2B). How genotype influences disease phenotype remains unclear and likely to be complex, probably depending on interactions among *GLA*, other potential modifying genes, environmental factors, and in the case of women, skewed X inactivation. The presence of other renal disease superimposed upon Fabry nephropathy may also modify prognosis. As late-onset FD sometimes involves both the renal and cardiovascular system [37], we cannot fully exclude concomitant latent cardiomyopathy since we did not perform endomyocardial biopsy in our patient. Besides, some organ involvement may be delayed in ‘later-onset’ phenotypes of FD. As our patient’s disease progresses, he may develop cardiac and cerebrovascular symptoms since he was only 36 years old at the most recent follow-up.

## Conclusion

We identified a novel deep intronic *GLA* mutation (IVS4+1326C>T) in a 33-year-old Chinese man with a mild α-GalA deficiency phenotype of Fabry podocytopathy and establishes a link between deep intronic *GLA* mutation and the renal variant of FD. Our research extends the mutation spectrum in *GLA* gene and justifies future studies of how IVS4+1326C>T and potentially other deep intronic *GLA* mutations contribute to Fabry podocytopathy through aberrant splicing. Future studies should assess the true incidence of the IVS4+1326C>T mutation in patients with different variants of FD, which may improve genetic diagnosis and personalized treatment.

## Supplementary Information

**Additional file 1**. Primer sequences for genomic DNA sequencing ,RT-PCR, qRT-PCR and PHAGE-*GLA* plasmid construction.

## Data Availability

Data cannot be made publicly available due to ethical reasons. In the area of rare diseases, information about the diagnosis in combination with personal information may compromise anonymity and confidentiality of the participants. The Ethics Committee from Xin Hua Hospital, Shanghai Jiao Tong University School of Medicine assessed our research project beforehand. The ethics vote allows sharing data with eligible researchers but we do not have approval to share the data publicly. Researchers interested in getting access to the data should feel free to contact the corresponding author (linfujun@xinhuamed.com.cn).
